# Efficacy and Safety of Bevacizumab Combined with Chemotherapy for Managing Metastatic Breast Cancer: A Meta-Analysis of Randomized Controlled Trials

**DOI:** 10.1038/srep15746

**Published:** 2015-10-27

**Authors:** Qin Li, Han Yan, Pengfei Zhao, Yifan Yang, Bangwei Cao

**Affiliations:** 1 Department of Oncology, Beijing Friendship Hospital, Capital Medical University, Beijing 100050, China

## Abstract

Although the FDA revoked metastatic breast cancer (MBC) from bevacizumab (BEV) indication in 2011, BEV combined with paclitaxel has been written in the breast cancer NCCN guidelines. This systematic assessment was performed to evaluate the efficacy and safety of BEV + chemotherapy (CHE) for managing MBC. PubMed and EMBASE were searched for original articles written in English and published before July, 2015. Progression-free survival was significantly improved in the CHE + BEV arms compared to the CHE arms in overall group and in human epidermal growth factor receptor 2-negative group (HR 0.75, 95% CI: 0.68–0.84, *P* < 0.001; HR 0.75, 95% CI: 0.69–0.82, *P* < 0.001). There were no significant improvement in overall survival in the CHE + BEV arms compared to the CHE arms. Significantly more grade 3 febrile neutropenia, hypertension, proteinuria, and cardiac events were observed in the CHE + BEV arm, which are controllable and reversible. Severe bleeding occurred more in the BEV + taxane arms and in patients with brain metastases. Therefore, CHE + BEV significantly increases progression-free survival in patients with MBC, it should be considered as a treatment option for these patients under the premise of reasonable selection of target population and combined CHE drugs.

Breast cancer is the most common type of malignant tumor in women worldwide. Metastatic breast cancer (MBC) is considered incurable, and the median survival at diagnosis is 2–4 years^1^. Molecular targeted therapy is the most active study in the various treatment options for MBC because it significantly improves survival, controls clinical symptoms, and maintains a favorable quality of life. Trastuzumab (TRA) is a humanized monoclonal antibody that is mainly used to treat patients with human epidermal growth factor receptor 2 (HER2)-overexpressing MBC. The addition of TRA to chemotherapy (CHE) was associated with longer survival (25.1 vs. 20.3 months, *P* = 0.01) and a 20% lower risk of death compared to patients who received CHE alone^2^. In patients with MBC, ado-trastuzumab emtansine statistically improved median overall survival (OS) by up to 5.8 months compared to lapatinib plus capecitabine (CAP)^3^. Pertuzumab and everolimus significantly prolonged progression-free survival (PFS) in patients with HER2-positive MBC^4^,^5^. Further large-scale studies of targeted therapies are being performed.

Bevacizumab (BEV) was the first anti-vascular endothelial growth factor (VEGF) monoclonal antibody to be approved by the United States Food and Drug Administration (FDA) to treat several tumor types. BEV inhibits tumor growth by preventing VEGF from binding to its receptor, thereby inhibiting tumor vascular endothelial cell proliferation and angiogenesis, reducing vascular permeability, and promoting tumor blood vessel degradation^6^. Unlike its use for colorectal cancer, lung cancer and kidney cancer, the use of BEV for MBC in the US has experienced numerous setbacks. The 2007 E2100 trial that showed that paclitaxel (PAC) plus BEV significantly prolonged PFS compared to PAC alone (11.8 vs. 5.9 months, *P* < 0.001) and increased the objective response rate (ORR) (36.9 vs. 21.2%, *P* < 0.001) led to the fast-track approval of BEV for MBC^7^. Because OS was not prolonged and there was an increased rate of adverse reactions in the BEV combination treatment group, the RIBBON-1 and AVADO trials met the FDA’s demand and provided further clinical data on PFS and OS. The RIBBON-1 trial confirmed that combining 15 mg/kg BEV with CAP or taxane/anthracycline-based CHE improved clinical outcomes by increasing PFS (8.6 vs. 5.7 months, *P* < 0.001; and 9.2 vs. 8.0 months, *P* < 0.001, respectively) compared to CHE alone^8^. However, the conclusion of the AVADO trial was not as positive because only the combination of docetaxel (DOC) with 15 mg/kg BEV, not 7.5 mg/kg BEV, resulted in a superior median PFS compared to the placebo (PLA) plus DOC regimen (PFS: PLA, 8.2 months; 7.5 mg/kg BEV, 9.0 months; 15 mg/kg BEV, 10.1 months)^9^. Because there was no significant improvement in clinical outcome, as had been expected, and the incidence of severe toxicity was higher, the FDA revoked its approval of BEV as a first-line treatment for MBC in 2011.

Although the clinical value of BEV in patients with MBC is controversial, it remains a promising strategy for treating MBC. A meta-analysis by Valachis confirmed that the addition of BEV to CHE evoked a meaningful improvement in PFS in patients with MBC^10^. For a more comprehensive and accurate understanding of the value of BEV in MBC, we expanded the number of included randomized controlled trials (RCTs) and performed a systematic assessment to analyze the efficacy and safety of BEV combined with CHE compared to CHE alone. We also performed a stratified analysis based on HER-2 status and the use of different CHE drugs in combination regimens with BEV, and we extensively discuss the possible reasons for controversy surrounding the use of BEV.

## Results

### Selection of RCTs

**Figure 1:**
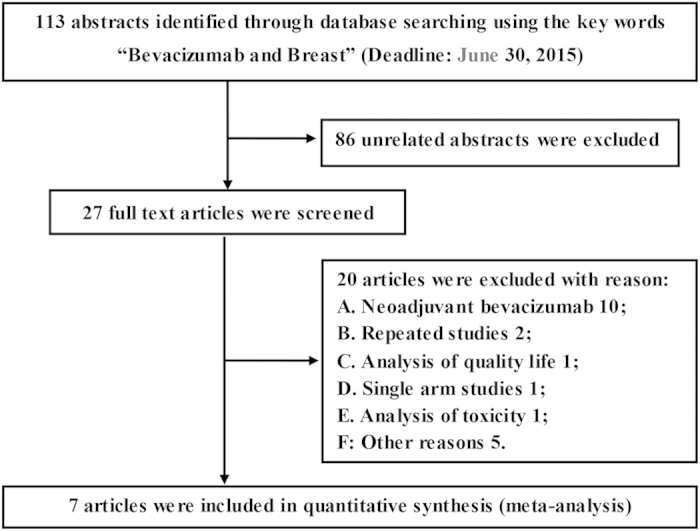
PRISMA flow diagram depicting the exclusion and inclusion of RCTs in the systematic assessment.

Figure 1 illustrates the inclusion and exclusion of studies in this systematic assessment^7^,^8^,^9^,^11^,^12^,^13^,^14^. In accordance with our search strategy, the database search identified 113 abstracts. Following the primary screening, 86 abstracts were excluded. Twenty of the remaining 27 full-text articles were excluded for various reason: they involved neoadjuvant BEV therapy, represented repeated or single-arm studies, or analyzed only quality of life or toxicity. A final set of seven articles was included in the quantitative synthesis. The PRISMA checklist can be found online in Supplementary Table S1.

### Risk of bias in the included studies

**Figure 2:**
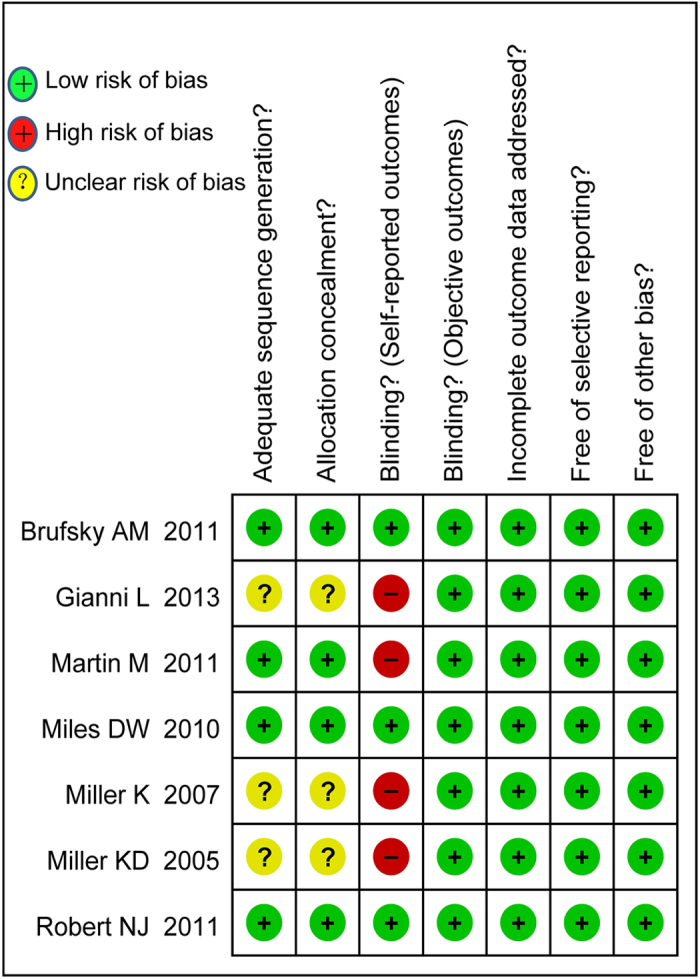
Risk of bias in the included studies.

Due to inadequate randomized sequence generation and inadequate allocation concealment, we assessed three RCTs as having an unclear risk of selection bias. Due to their open-label trial design, we assessed four RCTs as having a high risk of performance and detection bias (Fig. 2).

### Main characteristics of the studies included in the systematic assessment

Six trials were randomized phase III trials, and one was a randomized phase II trial. The primary endpoint was PFS in six trials and ORR in one trial. There were 4456 patients in this assessment, of which 2691 were included in the CHE + BEV group, and 1765 were included in the CHE group. There were 2848 HER2-negative patients in four trials and 1608 non-HER2-negative patients in three trials. There were 1312 patients with triple-negative breast cancer. Regarding treatment regimens, 1321 patients received BEV + CAP versus CAP, 1429 received BEV + DOC versus DOC, and 1126 received BEV + PAC versus PAC (Table 1).

### Efficacy analysis

**Figure 3:**
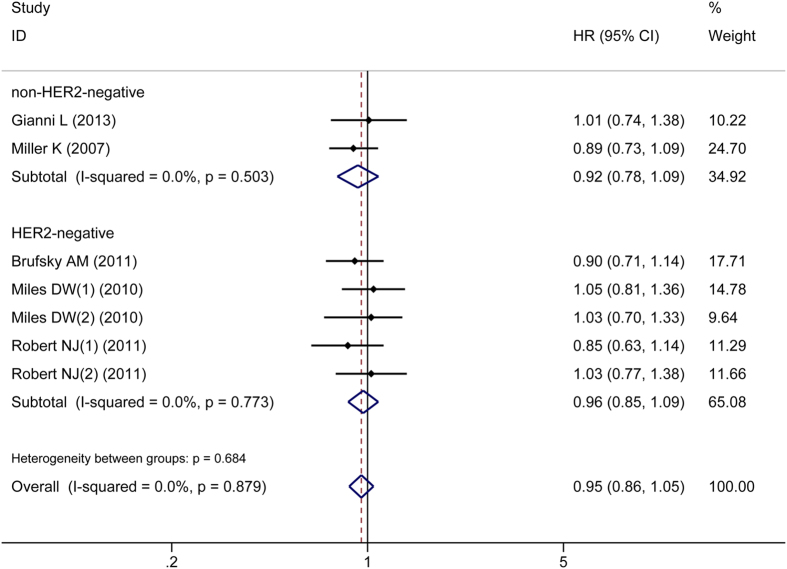
Comparison of OS between CHE + BEV and CHE.

OS, hazard risk (HR), and 95% confidence interval (CI) data were reported in four of the RCTs. One RCT reported only the OS and Kaplan–Meier curves; we used the Engauge Digitizer V4.1 (http://digitizer.sourceforge.net/) screenshot tool and a formula proposed by Parmar to estimate the HR and 95% CI for this study^15^,^16^. There was no significant heterogeneity in OS between the CHE + BEV and CHE groups (*P* > 0.05), so a fixed effects model was applied. The overall analysis indicated no significant improvement in OS in the CHE + BEV group compared to the CHE group (HR 0.95, 95% CI: 0.86–1.05, *P* = 0.313). Neither the HER2-negative subgroup nor the non-HER2-negative subgroup revealed a significant improvement in OS in the CHE + BEV group compared to the CHE group (HR 0.96, 95% CI: 0.85–1.09, *P* = 0.567; HR 0.92, 95% CI: 0.78–1.09, *P* = 0.355; respectively) (Fig. 3). Begg’s test and Egger’s test identified no significant publication bias (*Z* = 0.30, *P* = 0.764; *t* = 1.40, *P* = 0.221; respectively).

**Figure 4:**
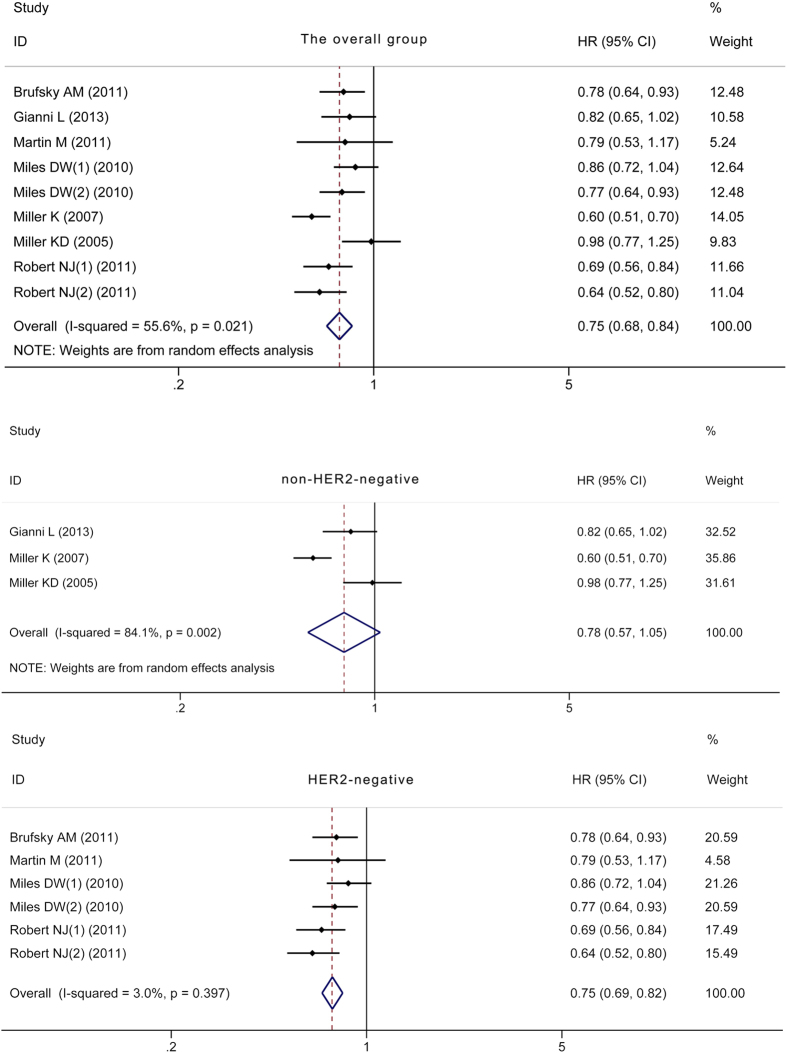
Comparison of PFS between CHE + BEV and CHE.

Complete PFS, HR and 95% CI data were reported in seven RCTs. There was significant heterogeneity in PFS between the CHE + BEV and CHE groups (*P* < 0.10) in the overall analyses and non-HER2-negative subgroup analyses, so a random effects model was selected. However, there was no significant heterogeneity in PFS between the CHE + BEV and CHE groups in the HER2-negative subgroup analysis (*P* > 0.10), so a fixed effects model was applied. The overall analysis and HER2-negative subgroup analysis indicated significantly improved PFS in the CHE + BEV group compared to the CHE group (HR 0.75, 95% CI: 0.68–0.84, *P* < 0.001; HR 0.75, 95% CI: 0.69–0.82, *P* < 0.001; respectively) (Fig. 4). The non-HER2-negative subgroup analyses did not yield similar results (HR 0.78, 95% CI: 0.57–1.05, *P* > 0.05) (Fig. 4). The PFS was significantly improved in the CHE + BEV group compared to the CHE group (HR 0.61, 95% CI: 0.47–0.80, *P* < 0.001) in patients with triple-negative MBC. Begg’s test and Egger’s test identified no significant publication bias in the overall, non-HER2-negative subgroup, and HER2-negative subgroup analyses (all *P* > 0.05).

**Figure 5:**
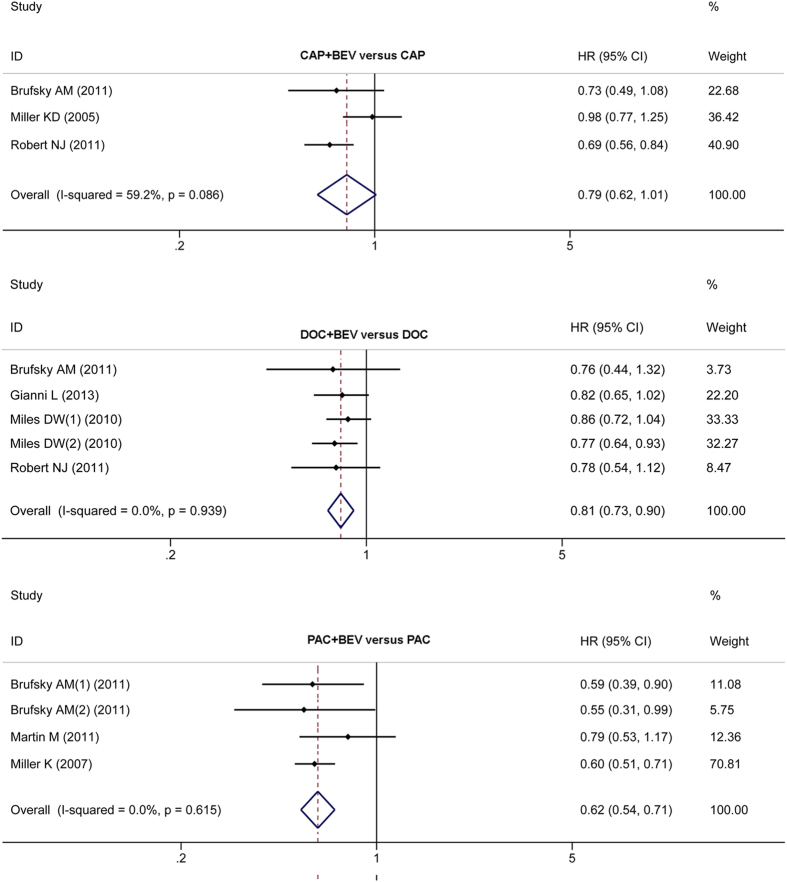
Subgroup comparison of PFS between CHE + BEV and CHE.

We performed subgroup analyses of the CHE (concrete) + BEV versus CHE (concrete) groups. PFS was significantly improved in the DOC + BEV versus DOC groups, and in the PAC + BEV versus PAC groups (HR 0.81, 95% CI: 0.73–0.90; HR 0.62, 95% CI: 0.54–0.71; respectively). Similar results was not obtained in the CAP + BEV versus CAP groups (HR 0.79, 95% CI: 0.62–1.01) (Fig. 5).

**Figure 6:**
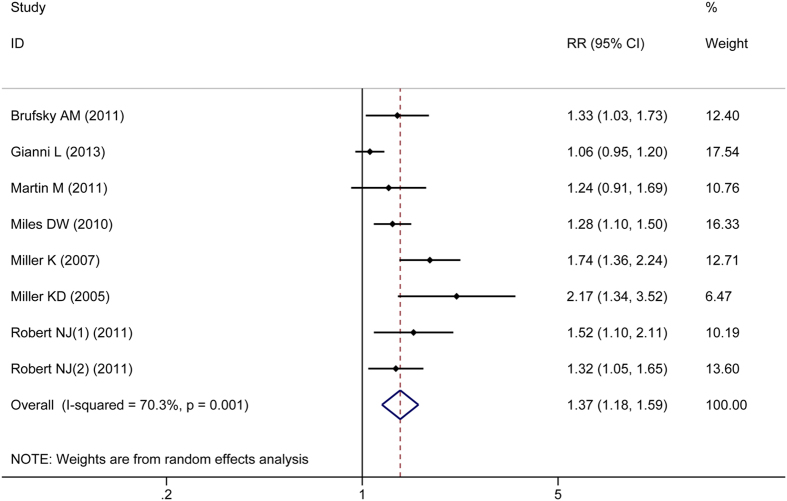
Comparison of ORR between CHE + BEV and CHE.

Complete ORR, HR, and 95% CI data were reported in seven RCTs. There was significant heterogeneity in ORR between the CHE + BEV and CHE groups in the overall analyses and non-HER2-negative subgroup analyses, so a random effects model was applied. However, the heterogeneity in ORR in the HER2-negative subgroup was not significant (*P* > 0.05), so a fixed effects model was applied. The overall and HER2-negative subgroup analyses indicated a significantly improved ORR in the CHE + BEV group compared to the CHE group (RR 1.37, 95% CI: 1.18–1.59, *P* < 0.001; RR 1.33, 95% CI: 1.20–1.47, *P* < 0.001; respectively) (Fig. 6). The non-HER2-negative subgroup analysis indicated no significant improvement in ORR in the CHE + BEV group (RR 1.54, 95% CI: 0.93–2.55, *P* = 0.090). Begg’s test and Egger’s test indicated no significant publication bias in the HER2-negative subgroup and non-HER2-negative subgroup analyses (all *P* > 0.05). Begg’s test revealed no significant publication bias (*P* = 0.108), while Egger’s test indicated significant publication bias (*P* = 0.013) in the overall population.

### Toxicity analysis

We extracted toxicity rates from all seven RCTs. There was no significant heterogeneity in the toxicity rates (*P* > 0.05) between the CHE + BEV and CHE groups, except for the incidence of hypertension, so a fixed effects model was applied. However, the heterogeneity for hypertension was significant (*P* > 0.05), so a random effects model was applied. There were significant increases in febrile neutropenia (≥Grade 3; RR 1.39, 95% CI: 1.08–1.80, *P* < 0.05), bleeding events (≥Grade 3; RR 4.33, 95% CI: 1.38–13.84, *P* < 0.05), hypertension (≥Grade 3; RR 7.15, 95% CI: 2.73–18.74, *P* < 0.01), proteinuria (≥Grade 3; RR 9.81, 95% CI: 3.76–25.58, *P* < 0.01), and cardiac events (≥Grade 3; RR 3.21, 95% CI: 1.47–7.01, *P* < 0.05). No significant increases in neutropenia (≥Grade 3), venous thromboembolic events (≥Grade 3), arterial thromboembolic events (any grade/≥Grade 3), gastrointestinal perforation (any grade/≥Grade 3), or cardiac events (≥Grade 2) were observed between the CHE + BEV and CHE groups (*P* > 0.05) (Table 2).

## Discussion

BEV is the most active targeted agent, and it significantly improves survival and controls clinical symptoms in many types of cancer. However, the role of BEV in MBC has been controversial.

BEV was approved in 2008 by the FDA for first-line treatment of HER-2 negative MBC in combination with PAC. In 2011, the FDA revoked MBC from BEV indication because of unexpected clinical outcome and the higher incidence of severe toxicity in BEV + CHE group. Nevertheless, BEV + PAC has been written in the breast cancer national comprehensive cancer network guidelines under the insistence of the experts. For a more comprehensive analysis of the efficacy and safety of BEV + CHE for managing MBC, the systematic assessment was performed.

Although some trials have achieved positive results in terms of PFS, others have reached opposite conclusions. Our overall analysis indicated a significantly improved PFS in the CHE + BEV group compared to the CHE group (HR 0.75, 95% CI: 0.68–0.84, *P* < 0.001), which is consistent with the conclusion by Valachis^10^. The analyses of the HER2-negative, triple-negative, DOC + BEV versus DOC, and PAC + BEV versus PAC subgroups yielded similar results to that of the overall analysis. From the point of view of PFS significant prolongation, BEV should be a treatment option for MBC patients in combination with CHE, especially in combination with PAC, DOC and in HER2-negative MBC patients.

The possible reasons that some studies did not observe an improved PFS are as follows: 1. BEV was used as a general therapy rather than as a targeted therapy and was not administered to patients with a specific molecular phenotype. 2. In the AVADO trial, the primary analysis determined that both 7.5 mg/kg BEV and 15 mg/kg BEV significantly improved PFS; however, an updated analysis determined that only the 15 mg/kg BEV arm experienced this benefit. Treatment assignments were not blinded following the primary data analysis, and potential investigator bias during tumor progression assessments may have negatively influenced the final result^9^. 3. The AVF2119g trial (CAP + BEV versus CAP) did not meet its primary endpoint of prolonged PFS (4.9 versus 4.2 months), but improved PFS was observed in the CAP cohort in the RIBBON-1 trial (8.6 versus 5.7 months)^8^,^14^, This latter positive finding suggests that the AVF2119g findings may have been due to the more heterogeneous nature of the study and to the higher number of patients with advanced MBC rather than from a lack of effectiveness of the combination therapy. 4. In the AVEREL trial, the investigator-assessed PFS HR was 0.82 (*P* = 0.0775), whereas the independent review committee-assessed PFS HR was 0.72 (*P* = 0.0162). The discrepancy between these two PFS values could have resulted from differences in imaging and lesion selection, the use of non-radiographic data, and clinical perceptions of new lesions^11^.

Despite the striking and promising improvements in PFS in these studies, the systematic analysis indicated no significant improvement in OS in the CHE + BEV group compared to the CHE group (HR 0.95, 95% CI: 0.86–1.05, *P* = 0.313). OS is considered the gold standard for clinical outcome; however, PFS has been used as an alternative endpoint to identify potential benefit at an earlier time point^17^. We found that the addition of BEV to CHE did not significantly prolong OS, but this by no means is a definite indication that BEV has no value in prolonging survival in MBC patients. The reasons for this conclusion may include the following: 1. PFS was designated as the primary endpoint in six of the RCTs, and ORR was designated as the primary endpoint in one RCT. According to statistical requirements, these study designs required an adequate number of patients to yield sufficient power to detect improvements in the median PFS or ORR. Therefore, these trials were not designed or adequately powered to detect differences in OS. 2. Many factors affect the final analysis of OS. After discontinuing their assigned treatment, the majority of patients received additional lines of treatment that included either CHE or hormonal agents, and patients were permitted to cross over from the CHE + PLA arm to the CHE + BEV arm^8^. Subsequent treatment data were not collected and analyzed for these patients, which may have compromised the ability to detect an improvement in OS^18^,^19^. Under such conditions, PFS better reflects the efficacy of BEV for the treatment of MBC than OS. 3. Treatment assignments were unblinded after the primary data analysis, which created the potential for investigator bias in tumor assessments, thereby potentially affecting the OS analysis^9^. 4. The AVADO trial results confirmed that the improvement in PFS was more pronounced in patients with high plasma VEGF-A concentrations after DOC + BEV treatment than in those with low VEGF-A concentrations^20^. Identifying and analyzing patient subgroups who show a BEV-specific molecular phenotype may encourage better survival. 5. The efficacy of BEV combination therapy may be affected by synergistic or antagonistic effects between BEV and different CHE regimens^21^,^22^. Therefore, the chemotherapeutic drugs that synergize with BEV are worthy of further research.

Treatment benefits and risks are equally important to patients, and the efficacy and safety of a drug are equally important in clinical trials. Under the premise that BEV + CHE significantly improves PFS, the safety of BEV determines its fate. Hamilton EP summarized the BEV safety data and arrived at the conclusion that BEV is generally well tolerated and that the majority of adverse events are mild and manageable^23^. Huang H’s meta-analysis revealed an increased risk of fatal adverse events in patients receiving BEV who had non-small cell lung cancer, pancreatic cancer, prostate cancer, or ovarian cancer. However, fatal adverse events were less common among breast cancer patients who were treated with BEV (RR0.61; 95% CI, 0.39–0.95)^24^. The fact that BEV has also been associated with certain severe toxicities should not be ignored, and these events should be reasonably analyzed to avoid their occurrence. Our meta-analysis indicated that severe neutropenia (≥Grade 3), venous thromboembolic events (≥Grade 3), arterial thromboembolic events (any grade/≥Grade 3), and gastrointestinal perforation (any grade/≥Grade 3) were infrequent and occurred at similar rates in the two arms. Severe febrile neutropenia, hypertension and proteinuria (all ≥Grade 3) were significantly more common in the BEV combination group, but these adverse events are controllable and reversible in clinical practice. With the exception of a higher rate 5.4% and 1.7% of bleeding complications in the BEV + taxane arm of the RIBBON-1^8^ trial and in the RIBBON-2 trial^12^, BEV + CHE does not significantly increase the incidence of serious bleeding (≥Grade 3). Patients taking anticoagulants or aspirin and those with treated CNS metastases, occult brain metastases or developing brain metastases were included in these trials, and this may partially explain the increased risk of serious bleeding^8^,^9^,^12^,^25^. Severe cardiac events (≥Grade 3) were apparently increased in the CHE + BEV group. However, the small number of events, including left chest wall radiation, left ventricular ejection fraction <50% at study entry, and pericardial metastatic involvement, prior to anthracycline exposure renders this comparison uncertain. To avoid severe toxicity, clinicians are required to carefully select patients and to avoid drug combinations that can lead to severe toxicity.

In summary, this systematic assessment indicates that CHE + BEV therapy confers clinical benefit in terms of increased PFS and ORR in patients with MBC, especially in HER2-negative MBC patients. Although CHE + BEV did not significantly improve OS, numerous influential factors in the study process dictate that we cannot simply dismiss the clinical value of BEV in OS. However, this combination therapy is associated with frequent adverse events. Thus, CHE + BEV should be considered as a treatment option for the patients with MBC under the premise of reasonable selection of target population and combined chemotherapy drugs. However, a major barrier to developing and implementing anti-angiogenic treatments is the difficulty in identifying patients who may benefit from BEV therapy. There is an urgent need to develop predictive molecular biomarkers that can guide patient selection and ensure the selection of optimal timing and the most synergistic chemotherapeutic drug combinations in patients with MBC. Large-scale, long-term follow-up studies will certainly reveal more answers.

## Methods

### Literature search strategy

We performed a systematic assessment according to Preferred Reporting Items for Systematic Reviews and Meta-analysis (PRISMA) criteria^26^. We searched PubMed, EMBASE, and the Central Registry of Controlled Trials of the Cochrane Library for original articles that were written in English and published before June 30, 2015. We also searched the annual meeting abstracts of the American Society of Clinical Oncology, European Cancer Conference, and European Society for Medical Oncology from the previous 15 years. To minimize the risk of selection or information bias, only prospective studies were included in our assessment. The initial search used the MeSH terms “Breast Neoplasm OR Neoplasm, Breast OR Breast Tumor OR Breast Tumors OR Tumor, Breast OR Tumors, Breast OR Breast Cancer OR Cancer, Breast OR Cancer of Breast OR Cancer of the Breast OR Breast Carcinoma OR Malignant Neoplasm of Breast OR Malignant Tumor of Breast” AND “Bevacizumab OR Avastin OR Genentech brand of Bevacizumab”. Additional search filters were “clinical trial” and “humans”.

### RCT selection and exclusion criteria

The following inclusion criteria were utilized: 1. The trial was prospective, properly randomized, controlled, well-designed, and matched for age, sex, tumor stage, and performance status or Karnofsky performance status. 2. Subjects were patients with MBC, and histological or cytological confirmation was required. 3. Control arm patients received CHE, CHE + PLA or CHE + TRA (collectively referred to as the CHE group); and experimental arm patients received CHE + BEV, CHE + PLA + BEV, or CHE + TRA + BEV (collectively referred to as the CHE + BEV group). 4. The endpoint was OS, PFS, ORR, and toxicity rates. 5. Explicit survival information or survival curves in the original article were presented as censored at last follow-up, with a follow-up rate of >95%. 6. Whenever trials with overlapping patient populations were encountered, we included only the trial with the longest follow-up.

### Data collection and extraction

Two investigators (Qin Li and Pengfei Zhao) independently assessed all the identified abstracts according to the predefined inclusion criteria. If only one investigator considered an abstract eligible, the full text of the article was retrieved, and both investigators reviewed it in detail. An arbiter (Han Yan) resolved any discrepancies, or the investigators contacted the authors of the original study. We extracted and evaluated the variables, including author names, journal, publication year, sample size per arm, performance status, treatment regimens, line of treatment, median patient age, sex ratio, tumor stage, and prespecified efficacy and safety outcomes.

### Assessment of methodological quality

Using the Cochrane Handbook for Systematic Reviews of Interventions^26^, the two investigators independently assessed the methodological quality of the included studies and resolved any disagreements by discussion. The investigators evaluated the risk of bias in the studies using Review Manager Software (RevMan Version 5.1, The Nordic Cochrane Center, The Cochrane Collaboration, Copenhagen, Denmark).

### Statistical analysis

We performed a systematic assessment using RevMan Version 5.1.7 (http://ims.cochrane.org/revman, The Nordic Cochrane Center) and Stata 11.0 (StataCorp LP, College Station, TX, USA). We investigated heterogeneity using Cochrane’s *Q*-test and *I*^2^ statistics. *P* > 0.1 and *I*^*2*^ < 50% indicated a lack of inter-study heterogeneity, and we calculated the pooled estimations of HR and risk ratio (RR) for each study using a fixed effects model (Mantel-Haenszel method). *P* < 0.1 and *I*^2^ > 50% indicated that the studies were heterogeneous, and we applied a random effects model (DerSimonian-Laird method). The principal measurements of effects were the HR and RR; these data are presented with a 95% confidence interval. All reported *P*-values are from 2-sided versions of the respective tests; *P* < 0.05 was considered statistically significant. Publication and selection bias were investigated through funnel plots using Egger’s test and Begg’s test^27^,^28^.

## Additional Information

**How to cite this article**: Li, Q. *et al.* Efficacy and Safety of Bevacizumab Combined with Chemotherapy for Managing Metastatic Breast Cancer: A Meta-Analysis of Randomized Controlled Trials. *Sci. Rep.* **5**, 15746; doi: 10.1038/srep15746 (2015).

## Supplementary Material

Supplementary Information

## Figures and Tables

**Table 1:** Main characteristics of the studies included in the systematic assessment.

Author	Trial	ER and/or PR+ or ER+/PR+%)	HER2+/− (%)	Cases (n)	Arms	Regimen
Gianni L	III	51.0	70.0/7.0	208	DOC + TRA	100 mg/m^2^ DOC + 8 mg/kg TRA loading dose followed by 6 mg/kg, IV, 3 weeks.
2013 [11]		53.0	71.0/9.0	216	DOC + TRA + BEV	100 mg/m^2^ DOC + 8 mg/kg TRA loading dose followed by 6 mg/kg; 15 mg/kg BEV, IV, 3 weeks.
Robert NJ	III	73.7	0.0/95.1	206	CAP + PLA	1000 mg/m^2^ CAP twice daily for 14 days, PO, 3 weeks.
2011 [8]		77.4	0.0/95.8	409	CAP + BEV	1000 mg/m^2^ CAP twice daily for 14 days, PO; 15 mg/kg BEV, IV, 3 weeks.
		76.9	0.0/99.0	207	TAX/Anthra + PLA	TAX-based (260 mg/m^2^ nab-PAC, 75–100 mg/m^2^ DOC) or Anthra-based (DOX or EPI combinations [DOX/CTX, EPI/CTX, FU/EPI/CTX, or FU/DOX/CTX]); 15 mg/kg PLA, IV, 3 weeks.
		76.1	0.0/98.8	415	TAX/Anthra + BEV	TAX-based (260 mg/m^2^ nab-PAC, 75–100 mg/m^2^ DOC) or Anthra-based (DOX or EPI combinations [DOX/CTX, EPI/CTX, FU/EPI/CTX, or FU/DOX/CTX]); 15 mg/kg BEV, IV, 3 weeks.
Brufsky AM 2011 [12]	III	73.3	0.0/85.3	225	CHE + PLA	1000 mg/m^2^ CAP twice daily for 14 days, PO, 3 weeks; 75–100 mg/m^2^ DOC, IV, 3 weeks; 260 mg/m^2^ nab-PAC, IV, 3 weeks; 90 mg/m^2^ PAC, IV on D1, D8, D15, 4 weeks or 175 mg/m^2^, IV, 3 weeks; 1250 mg/m^2^ GEM, IV, D1, D8, 3 weeks; or 30 mg/m^2^ VIN, IV, 3 weeks. 10–15 mg/kg PLA, IV, 2–3 weeks.
		71.7	0.0/83.9	459	CHE + BEV	1000 mg/m^2^ CAP twice daily for 14 days, PO, 3 weeks; 75–100 mg/m^2^ DOC, IV, 3 weeks; 260 mg/m^2^ nab-PAC, IV, 3 weeks; 90 mg/m^2^ PAC, IV on D1, D8, D15, 4 weeks or 175 mg/m^2^, IV, 3 weeks; 1250 mg/m^2^ GEM, IV, D1, D8, 3 weeks; or 30 mg/m^2^ VIN, IV, 3 weeks. 10–15 mg/kg BEV, IV, 2–3 weeks.
Martin M	II	80.0	0.0/100	94	PAC + PLA	90 mg/m^2^ PAC, D1, D8, D15, IV, 3 weeks; masked placebo orally once daily.
2011 [13]		80.0	0.0/100	97	PAC + BEV	90 mg/m^2^ PAC, D1, D8, D15, IV, 3 weeks; 10 mg/kg BEV, D1, D15, IV, 4 weeks.
Miles DW	III	78.0	0.0/100	241	DOC + PLA	100 mg/m^2^ DOC, D1; 7.5–15 mg/kg PLA, IV, D1, 3 weeks.
2010 [9]		78.0	0.0/100	248	DOC + BEV (7.5 mg)	100 mg/m^2^ DOC, D1; 7.5 mg/kg BEV, IV, D1, 3 weeks.
		76.0	0.0/100	247	DOC + BEV (15 mg)	100 mg/m^2^ DOC, D1; 15 mg/kg BEV, IV, D1, 3 weeks.
Miller K	III	62.9/45.1	0.9/89.9	354	PAC	90 mg/m^2^ PAC, D1, D8, D15, IV, 4 weeks.
2007 [7]		59.9/44.7	1.4/92.5	368	PAC + BEV	90 mg/m^2^ PAC, D1, D8, D15; 10 mg/kg BEV, D1, D15, IV, 4 weeks.
Miller KD	III	51.7/41.7	20.4/unknown	230	CAP	1250 mg/m^2^ CAP twice daily for 14 days, PO, 3 weeks.
2005 [14]		41.7/32.3	26.3/unknown	232	CAP + BEV	1250 mg/m^2^ CAP twice daily for 14 days, PO; 15 mg/kg BEV, IV, D1, 3 weeks.

**Table 2:** Comparison of toxicity rates between CHE + BEV and CHE regimens.

	n/N (CHE + BEV)	n/N (CHE)	I^2^ (%)	P	RR	95% CI	P
Neutropenia (≥Grade 3)	263/2320	183/1670	0.0	0.658	1.03	0.91–1.29	0.393
Febrile neutropenia (≥Grade 3)	141/2213	83/1555	0.0	0.765	1.39	1.08–1.80	0.012
Bleeding events (≥Grade 3)	25/1844	2/1183	8.0	0.361	4.33	1.38–13.84	0.012
Hypertension (≥Grade 3)	262/2417	23/1919	72.1	0.000	7.15	2.73–18.74	0.000
Proteinuria (≥Grade 3)	62/2521	1/1852	0.0	0.991	9.81	3.76–25.58	0.000
Venous thromboembolic events (≥Grade 3)	41/1490	33/1038	0.0	0.494	0.80	0.50–1.28	0.356
Arterial thromboembolic events (any Grade)	13/1019	6/603	0.0	0.865	1.29	0.49–3.40	0.604
Arterial thromboembolic events (≥Grade 3)	5/952	3/680	43.3	0.184	0.96	0.27–3.37	0.946
Gastrointestinal perforation (any Grade)	11/1021	5/604	0.0	0.673	1.69	0.57–4.96	0.342
Gastrointestinal perforation (≥Grade 3)	5/1317	4/1025	0.0	0.407	0.94	0.29–3.04	0.917
Cardiac events (≥Grade 2)	31/1015	9/609	0.0	0.617	1.97	0.95–4.08	0.067
Cardiac events (≥3 Grade)	28/1605	7/1317	0.0	0.426	3.21	1.47–7.01	0.040
